# Effects of Plyometric and Repeated Sprint Training on Physical Performance

**DOI:** 10.3390/sports8070091

**Published:** 2020-06-27

**Authors:** Ivan Krakan, Luka Milanovic, Ivan Belcic

**Affiliations:** Faculty of Kinesiology, University of Zagreb, 10000 Zagreb, Croatia; ivan.krakan@kif.hr (I.K.); luka.milanovic@kif.hr (L.M.)

**Keywords:** repeated sprint ability, plyometric training programs, performance, sprint, football training

## Abstract

The purpose of study was to resolve the effect of plyometric training and repeated sprint training on physical performance. The study was conducted on 41 subjects in two experimental groups (plyometric/repeated sprints training). Before and after the training program, subjects were subjected to diagnostic procedures that included standard test protocols. Results proved a statistically significant difference only after the plyometric training program compared to the repeated sprint group in countermovement jump (8.65% vs. 2.21%). In variable repeated jumps, an increased value was recorded (2.9% vs. 4.29%), like in sprint variables after the training program where certain trends of progress happened after the repeated sprint ability training and the specificity of the program (5 m = 0.89%, 10 m = 1.07% and 25 m = 1.35%), while plyometric training recorded unchanged values at 5 and 10 m, and a 0.27% improvement at 25 m. Stagnation of the 20-yard test was recorded in both groups. There was no difference between training programs in any variables of functional capacities, with similar measures recorded in repeated sprint ability. After six weeks of both training types, positive changes can be expected in explosive strength of lower extremities, increases in acceleration area, and maximum speed.

## 1. Introduction

The dynamics of sports games imply the constant repetition of high-intensity (submaximal) or maximal activities intersected by short periods of low-intensity activities or rest that are repeated continuously throughout the match or encounter [1]. Sprinting represents one of the key categories of movement in team sports [2], and this ability diminishes as a result of fatigue as the match or competition progresses [3]. The ability to resist fatigue and maintain the maximum intensity of activity throughout the match or encounter has proven to be a very important ability for team sports athletes. This ability is called repeated sprint ability (RSA). The use of notation analysis and the increasing use of GPS (global positioning system) in team sports games (football, rugby, basketball, hockey) have determined the characteristics of the moving structures that appear in these sports, as well as the occurrence of sprints. Football is an acyclic and intermittent sport, where short-lived high-intensity activities, such as 20 m sprints (sprint is defined as running above 25 km/h) and high-intensity actions such as counter-attacks, are intertwined with low and medium intensity activities (fast walking and jogging), and rests such as standing. Although the total distance covered by players during the match has not changed significantly over the years, the demands for high intensity running and longer sprint distances during the match have changed [4]. Using motion analysis, it has been noted that a relatively large number of sprints are realized during matches in sports games, and that greater distance achieved by sprinting differentiates the top teams [5]. In addition, statistically significant decreases in sprint distance were found as well as number of sprints and the percentage of time spent sprinting in the second half compared to the first half. The greatest declines in sprint distance were found in lower quality teams [6]. In professional football players, there is a significant correlation between the average sprint time in the repeated sprint ability test and the distance covered by high intensity running (r = –0.65) and sprint (r = –0.6) [7]. The repeated sprint training program presents repetitions of straight or return sprints on a short section with a recovery time of less than 60 s between repetitions. Repetitive sprint training is a training method that is specific to the development of this ability [1,8], and the authors consider that this improvement is a result of the specificity of the training process itself [9]. 

Given the demands that team sports put on athletes, the application of strength training programs, explosion training, and plyometric training are technologies that we often encounter during the athletic preparation period. Plyometric training is a popular form of work on physical conditioning of healthy individuals and has been the subject of much research in the last three decades. The available literature [10] states that plyometrics, alone or in combination with other training modalities, produces numerous positive changes in the space of the nervous and musculoskeletal systems, muscle function, and performance in healthy individuals. In team sports such as basketball, handball, volleyball, netball, or Australian football, players must repeat consecutive explosive activities, such as short sprints (from under 5 m until 25 m) with frequent changes of direction [2,11,12], followed by activities such as maximal jumps. In addition, jumps occur mostly after high intensity/sprinting activities, such as vertical jumps after a quick attack in handball [13]. Other studies support the claim that performance improvement in motor-specific tasks such as vertical jumping, long jump, maximum running speed, as well as running economy can be expected with the use of plyometric training [14,15,16].

The main aim of this paper is to determine the impact and differences of plyometric training and repetitive straight-line sprint training on motor abilities, functional abilities, and repeated sprint ability.

## 2. Materials and Methods

### 2.1. Ethics Committee Approval

This study has been approved by the ethics committee of the Faculty of Kinesiology, University of Zagreb.

### 2.2. Subjects

The subjects were students (41) of the first year in the Faculty of Kinesiology, University of Zagreb and were divided into two experimental groups. All subjects were active athletes from the amateur to semi-pro level. The first group of subjects (181.23 ± 6.92 cm, 80.54 ± 8.12 kg) conducted repeated straight-line sprint training, and the second group (175.36 ± 6.19 cm, 77.29 ± 9.50 kg) conducted plyometric training. When engaging in the experimental process, the subjects were asked not to take any special supplements that can increase their performance and not to carry out any additional form of physical exercise, while the basic form of physical exercise during the experiment was a practical class on Faculty. All subjects were informed of the purpose of the research and the possible risks of participating in the study prior to conducting it and confirmed their agreement by signing the voluntary consent to participate in the experiment.

### 2.3. Variable Samples

- Repeated straight-line sprint ability (RSA)—6 × 25 m with a start every 25 s
- The explosive power—jump type
  ○ A countermovement jump—CMJ (cm)
  ○ Repeated Jumps—RJ (cm)
- The explosive strength—sprint type
  ○ 5 m—SP5 m
  ○ 10 m—SP10 m
  ○ 25 m—SP25 m
- Agility test—MAG20y (s)
- Best straight-line sprint result—RSAb (s)
- Average straight-line sprint results—RSAp (s)
- Percentage of decline in straight-line sprint performance during the test—RSA%Sdec (%)
  ○ Calculated using [13], the equation %Sdec = 100 – RSAp/RSAb × 100
- Concentration of lactates after repeated straight-line sprint test after 3 min—RSA_La (mmol/L)
- A subjective load rating (RSA_RPE)
- Evaluation of functional abilities—progressive spiroergometric load test on a treadmill
  ○ Maximum oxygen uptake—VO_2_max
  ○ Maximum speed of treadmill—vmax
  ○ Maximum speed of treadmill at maximum oxygen uptake—vVO_2_max
  ○ Maximum heart frequency—Fsmax
  ○ Peak heart frequency during repeated straight-line sprint test—FSpeak_RSA
  ○ Heart rate frequency 1 min after repeated straight-line sprint test—FS1min

### 2.4. Procedure

At the Faculty of Kinesiology, University of Zagreb Diagnostic Center, participants were subjected to measurement of anthropometric characteristics. For defining the morphological status of the subjects, variables of body height (cm) and body weight (kg) were measured. A set of eight tests was used to test physical abilities, of which seven were chosen to test motor skills, while one was used to assess functional abilities of subjects.

Repeated straight-line sprint ability (RSA) was tested as 6 × 25 m sprint with a start every 25 s. In this test best sprint (RSAb) (s), average of sprints (RSAp) (s), and percentage of decline of sprint performances during the test (RSA%dec) (%) were calculated using the equation RSA%dec = 100 – RSAp/RSAb × 100. For every subject, concentration of lactates (RSA_La) and subjective load estimation (RSA_RPE) was measured after repeated straight-line sprint test.

Explosive strength—sprint type was tested with a 25 m sprint test with time intervals at 5 m, 10 m, and 25 m with 2 min of passive rest between sprints. An average value of 3 sprints was included in the analysis.

The explosive power—jump type was tested with the Microgate Optojump Next system to measure the height of the reflection, the duration of contact with the ground, as well as the duration of the flight phase. Countermovement jump—CMJ (cm) was performed in the starting position with the subject’s hands isolated on the hips and subject’s upright standing position for a few seconds. The subject then lowered into a semi-squat to an approximate angle of 90 degrees between the upper leg and lower leg. From the descent, without stopping, the subject performed a maximum vertical jump, and then landed with a slight flexion in the knees. The test was repeated three times and the average value of the three results was included in the analysis. In the test of repeated jumps, the subject’s hands were isolated on the hips and the subject stood in an upright position outside a measured space. At the sound of a laptop, using system software, the subject jumped into the measurement space and performed 6 consecutive jumps from the feet, without bending at the knee joint when in contact with the surface. As a result, the average value of the height of these jumps was taken.

A Witty photocell telemetry system was used to test agility in a 20-yard test, which had two parallel lines 10 yards apart with 1 line placed exactly in the middle. From the position of the high start, the subject started the test on a personal signal, which he repeated three times. The subject moved to the side line by touching the line with his foot (exceeding a distance of 5 yards), then sprinted to the other side line where he had to also touch with his foot (exceeding a distance of 10 yards) and finally sprinted to the starting, center line where time was stopped. The average value of the three results was taken as the result.

A spiroergometric progressive load test on a treadmill (HC1200, Technogym, Gambettola, Italy) was used to test functional abilities. The test was conducted in a closed and ventilated laboratory with constant standard microclimatic conditions (19–21°C). The subject breathed with the nose and mouth through a breathing mask (Hans Rudolph, Shawnee, KS, USA), which was connected to a bidirectional turbine with an optocoupler airflow reader. A sample of air (1 mL/s) was pulled out of the turbine through a Nafion Permapure capillary tube (removing moisture without influencing the gas concentration) to fast oxygen (zirconium) and carbon dioxide (infrared) analyzers. Ventilation–metabolic parameters were monitored for each breath-by-breath cycle and displayed numerically and graphically on a monitor during real-time testing. The test protocol started with the subject relaxing on a treadmill, and after one-minute, the subject started to walk at a speed of 3 km/h for 2 min at a constant incline of 1.5%. After every 30 s, speed was increased by 1 km/h until the subject was unable to follow the increases in speed. 

All tests were performed in the hall of the Faculty of Kinesiology according to standard measurement protocols on a hard surface and with constant microclimate conditions. The study program lasted for a total of nine weeks with initial testing in the first week and introduction trainings (2 times 60 min) in the second week. The experimental program lasted in total six weeks (from the third week until the eighth week) with 3 training sessions per week lasting 60 min each. Research was finished in the ninth week of the program with final tests. 

The experimental group conducting plyometric training (Table 1) performed unilateral and bilateral jumps in the vertical and horizontal directions. The number of sets ranged from 1 to 3 per training session with 120 jumps in a single practice (385 jumps at the end of the week), up to a maximum of 180 jumps per training session (total of 550 jumps per week). The rest between sets ranged from 30 s to 1 min of passive rest, and between types of jumps there was two minutes of passive rest. The experimental group, which conducted repetitive sprint training (Table 2), had the number of sprints range from 6 sprints in a set in the first week up to 10 sprints in the set in the last week, with two to three sets per training. The sprints were conducted on a 20 m section with passive rest of 25 s between repetitions and passive rest of two minutes between sets.

### 2.5. Data Analysis

Statistical processing software Statistica for Windows version 13.4 (StatSoft, Inc., Tulsa, OK, USA) was used for data processing. The arithmetic mean and standard deviation were calculated for all measured parameters, while the normality of the distributions was tested by the Kolmogorov Smirnov test. Effect size was calculated through partial eta squared. Variance analysis for repeated measures (2 × 2 ANOVA) was used to analyze differences between groups after 78 training programs. Additionally, the main effects and interactions among experimental groups were measured. The statistical significance level of the differences was tested at the 0.05 level.

## 3. Results

### 3.1. Effects of Plyometric Training and Repeated Sprint Training on Motor Skills and Repeated Sprint Ability

Univariate analysis (Table 3) of the initial state showed that there were no statistically significant differences between the groups in the initial measurement in the space of explosive strength—jump type. Plyometric training and repeated straight-line sprint training programs resulted in a significant improvement in the results of countermovement jump, and there were differences between the groups after the training process. Analyzing the magnitude of the effect of treatments (plyometric training and repeated sprints) on physical fitness through partial eta square obtained through univariate analysis of results, large effects in the explosive strength of the lower extremities were obtained (0.25). Mean training effects were obtained in the RSAb and RSA_RPE variables within groups between the final and initial measurements, but no differences were obtained in the magnitude of treatment effects between groups.

### 3.2. Effects of Plyometric Training and Repeated Sprint Training on Functional Abilities

After repeated straight-line sprinting and plyometric training, using univariate analysis of variance (Table 4) for repeated measurements, there were no statistically significant differences between the experimental groups in the measured variables (VO_2_max-*p* = 0.737, vmax-*p* = 0.06, vVO_2_max-*p* = 0.749, FSmax-*p* = 0.141, FSpeak_RSA-*p* = 0.128). Certain differences in aerobic capacity were obtained in both experimental groups, and there was a positive trend in the repeated sprints program experimental group, but no statistically significant differences were obtained between the groups after the training procedure. Experimental training programs led to statistically significant changes in the final measurements according to initial measurements of the following variables: the maximum speed on the treadmill of 3.6% in the group that performed repeated sprints training versus the 1% of plyometric group (*p* = 0.002); maximum heart rate progress achieved in the progressive load test (*p* = 0.019) by repeated sprint ability group was 1.75%, while other training group had 0.42%); the peak heart rate during the repeated sprint test (*p* = 0.000005) with an improvement of 5.16% compared to the plyometric group (2.66%); and heart rate frequency one minute after repeated straight-line sprint test (*p* = 0.0006) with 6.71% in the repeated sprint ability training group and with 2.81% in the plyometric training group.

## 4. Discussion

### 4.1. Effect of Plyometric and Repeated Sprints Training on Motor Abilities

A statistically significant difference was found in the variable countermovement jump in the group that conducted plyometric training (8.35%). In the group that conducted the training of repeated straight sprints, this progress was lower (2.21%). After the process with the plyometric training group, unchanged values were recorded at 5 and 10 m, while at the 25 m section, the development trend indicated positive effects of treatments, although they were not statistically significant (0.27%). The treatment of repeated straight-line sprints achieved an improvement in results in the final measurements at 5, 10, and 25 m by 0.89%, 1.07%, and 1.35%, respectively. The improvement in height of the countermovement jump was in line with previous studies that reported progress [17,18]. It is concluded that increases in results after short plyometric training (6–16 weeks) was due to changes in tension of elastic components of plantar flexor tendons, increased agonist muscle nerve impulse, improved inter-muscular coordination, changes in muscle size and architecture, and changes in the mechanisms in the individual muscle fiber. One of the reasons why plyometric training affects the values of results in the countermovement jump compared to repetitive sprint training is the ability of plyometric training to cause hypertrophic effects. In a meta-analysis [10], the authors stated that 6 to 8 weeks of plyometric training causes a greater cross-section of type I (23%), type IIa (+ 22%), and type IIb (30%) muscle fibers in vastus lateralis muscle, which can also be associated with greater strength and explosive power of the knee extensor and can result in higher values in the countermovement jump variable. In addition, one of the reasons is the maximal voluntary muscular contraction and action of plantar flexors, which is a consequence of the activation of more motor units. Improvement of intramuscular coordination is cited as one of the explanations for neural adaptations to plyometric training, the coordination of the pre-landing phase, and changes in the elongation of the myotatic reflex. Furthermore, plyometric training is associated with a greater amount of power produced (3–5%) in the leg extensors, which is accompanied by a larger cross-section of vastus lateralis and type I (4.4%) and type II (7.8%) muscle fibers. Plyometric training resulted in changes in the strength and power of the musculature based on the neural and muscular changes that occurred in the individual’s body. Minor training effects in jump type explosiveness were evident in the group that performed repetitive straight-line sprint training, although this mode caused great neuromuscular effort and increased jump type—explosiveness [14]. More progress may be prevented due to the work-to-rest ratio of the group that performed straight-line sprint training. One of the reasons may be the specificity of the individual’s adaptation and the transfer of that training to the final result as a consequence of plyometric training on the height of the jump, as the training of repeated sprints resulted in better values in the test of repeated sprints. In the 20-yard test, stagnation of results was found in both groups. Similar results were obtained by a group of authors [19] where they did not record improvements in the three agility tests. Although it was expected that plyometric training would result in a greater improvement in ability to change direction due to a similar muscle regimen that resembles a stretching and shortening cycle, it did not happen. The task of agility may depend more on motor control than muscle strength and power, which may be one of the reasons why stagnation of progress in agility space has been reported. After the experimental training programs, some trends in the development of explosive power—sprint type could be noticed. After plyometric training, the 5 and 10 m sections recorded unchanged values. On the 25 m section, the results showed positive increases by 0.27%. In previous studies where the training program lasted longer than 6 weeks [18,20,21], there was an improvement and a trend of improvement in results after the plyometric program, while in the programs lasting 6 weeks or shorter, there were none. The frequency and volume of training are crucial parameters in the choice of positive adaptations of plyometric training to sprinting ability [22]. Such plyometric training duration of less than 10 weeks (6–8) with a frequency of 3–4 trainings per week resulted in better values. A training program that had more than 18 training units with more than 80 jumps represented the optimal volume (like in this study: 18 training units, 120–185 contacts with the surface). One of the reasons why there was no major change in the sprint in the 5, 10, and 25 m sections was the short total training volume of six weeks as well as the number of training units in it and the large volume of jumps per individual training. This increased values of fatigue in individuals after the training sessions and a large volume of training in a short period. The benefits of plyometric training on sprints were most expected in this phase, which was closely linked to the abilities—the acceleration phase, but this was not realized. The sprint improvement on the overall sprint section of 25 m was higher in the group that performed repeated straight-line sprint training (1.35% vs. 0.27%). This difference was statistically not significant and could be explained by the relationship between extensor strength and power of the lower extremities.

### 4.2. Effect of Plyometric and Repeated Sprints Training on Functional Abilities

Repetitive sprint training is a training technology that greatly affects the neuromuscular system, and a review of recent studies shows that repetitive sprint training has had positive effects on the functional abilities displayed by maximal oxygen uptake [5,8,23], although the stimulus applied is very short. The basic prerequisite for a high level of aerobic fitness in athletes is the value of oxygen uptake, the highest speed achieved on a treadmill during the progressive load test, lactate threshold, and speed at the lactate threshold. However, in homogeneous groups of athletes where the levels of maximum oxygen uptake and other variables are quite similar, other parameters began to be considered such as running technique—economy [10]. Referring to other study [24], the same authors cite improvements in running economy contributing to power values of 3.1% over time at 5 km, while the values of maximum oxygen uptake are decreased by 5.8% due to plyometric training (in long-distance runners 1.2% improvement in time but also a 3.1% decrease in maximum oxygen uptake). The reasons for improving the economy of movement are not yet fully known, although the emphasis is on improving the activation of motor units and reducing contact time with the surface during movement. The biggest changes in progressive load test indicators after treatment of plyometric training were recorded by experimental groups that conducted plyometric training in a unilateral manner and with a horizontal direction of action. They were 13% better than the groups that applied vertical or a combination of vertical and horizontal jumps. The reasons for effects of plyometric training on the final results in progressive load tests could be explained by reduced contact time with the surface, improved tendon and muscle rigidity, increased mechanical output caused by the elastic attributes of the muscles and tendons, and better movement economy as a whole. The maximum heart rate decreased by 0.5% in the plyometric training group. In the group that performed the repeated sprint training, it decreased by 1.8%. Decrease in reactivation of the parasympathetic part of the autonomic nervous system is greater after repeated sprint training than after moderate continuous aerobic training, and the reason is attributed to the anaerobic component of activity, which is significantly represented in repeated sprint training, which leads to prolonged activity of the sympathetic part of the autonomic system when establishing hormonal homeostasis [25]. The group of repeated straight-line sprints recorded a significant improvement in maximum running speed on a progressive load test with percentage of 3.6%, and this value remained unchanged in plyometric training. Although a positive trend can be seen, there were no statistically significant changes in vmax and vVO_2_max after the training program, and it is difficult to expect major changes in lactate concentration after the test. Metabolite accumulation increases beyond the anaerobic threshold and further after exceeding the critical intensity, as a greater metabolic reaction occurs after the passage of vVO_2_max. After the training program, the vVO_2_max and vmax values did not change significantly, and there was no significant change in lactate concentration after treatment, although trends of changes could be seen. The absence of changes after plyometric training can be attributed to the reduced involvement of the metabolic system in the realization of the training program as well as the rest intervals between repetitions and series in the training program.

### 4.3. Effect of Plyometric and Repeated Sprints Training on Repeated Sprint Ability

The effects of the treatment between the two experimental groups in the 6-week plyometric training and repeated straight-line sprint training programs did not contribute to statistically significant differences in most variables of repeated sprint tests. Repeating straight-line sprint training resulted in an increase in value (2.1%) of RSAb after the training program as well as plyometric training (1.6%), while no differences were obtained between the groups after the training program. RSAp recorded unchanged results in the final compared to initial testing, while RSA%Sdec recorded an improvement of 23% in repeated straight sprint training and 22.1% in the plyometric training group, with no differences between the groups. Comparing the results of previous studies [13,26] with the results obtained in this study, it can be seen that the progress of the group that performed the repetitive sprint training was lower in the variables RSAp, RSAb, and RSA%Sdec than those reported in previous studies [27], while on the other hand, a similar trend was obtained in the RSAb and RSAp variables [26,28]. The unchanged values in the RSAb and RSAp variables in both experimental groups are logical with already proved high correlation between maximum sprint and sprint averages [9]. A reason for the absence of changes may be the training status of the first-year students of the Faculty of Kinesiology, who represent the active part of the population, unlike in a study of a group of authors [22] who found larger changes in less experienced individuals.

## 5. Conclusions

The program of repeated straight-line sprints and the program of plyometric training did not produce statistically significant differences in most variables in the area of motor abilities. A statistically significant difference was obtained in the variable countermovement jump in the plyometric training group (8.65%), while in the other variables, there was a certain trend of improvement in individual abilities after 6 weeks of plyometric training and repeated straight-line sprint training. Straight-line sprint and plyometric training programs cause similar changes in the functional abilities measured by a progressive load test, with no differences in abilities between groups after the training program. Both training protocols resulted in similar values in repeated sprint abilities with unchanged results in the fastest sprint in the test. In addition, average of sprints during the test was unchanged, while values in the sprint declines recorded improvements. The results of this study indicate that after six weeks of plyometric training and repetitive straight-line sprint training, changes in the explosive strength of the lower extremities can be expected in the plyometric training group, with also observed trends of improvement in acceleration space and maximum speed. Subjective load assessment recorded statistically significantly lower values in both groups after training, and the conclusion is that both groups increased their fatigue tolerance after the training program. Analyzing the percentages between groups in the effects of training, plyometric training resulted in higher values in the area of explosive strength compared to the training of repeated sprints. 

## Figures and Tables

**Table 1:** Plyometric training experimental group’s 6 week training program.

Week		1			2			3			4			5			6		
Training		1	2	3	4	5	6	7	8	9	10	11	12	13	14	15	16	17	18
**Exercises**		Number of sets/number of repetitions (rest after set)—rest after exercise 1′–2′																	
**Bilateral:**	jumps from the feet in vertical movement	3/10 (30″)	3/10 (30″)	2/10 (30″)	2/10 (30″)	2/10 (30″)	2/10 (30″)	2/10 (30″)	2/10 (30″)	2/10 (30″)	1/10	1/10	1/10	1/10	1/10	1/10			
	semi-squat jumps in vertical movement	3/10 (30″)	2/10 (30″)	2/10 (30″)	2/10 (30″)	2/10 (30″)	2/10 (30″)	2/10 (30″)	2/10 (30″)	1/10 (30″)	2/10 (30″)	2/10	2/10	1/10			1/10	1/10	1/10
	semi-squat jumps in horizontal movement		1/10 (30″)	3/5 (30″)	3/5 (1’)	3/5 (1″)	3/5 (1″)	1/5	1/5 (1’)	2/5 (1’)	2/5 (1’)	1/5	1/5	1/5	1/5	1/5	2/5 (1’)	2/5 (1’)	2/5 (1’)
**Contacts—Bilateral jumps**		60	60	55	55	55	50	45	45	40	40	35	35	25	15	15	20	20	20
**Unilateral:**	jumps from the feet (L/R) in vertical movement	2/5 (45″)	2/5 (45″)	2/5 (45″)	3/5 (45″)	3/5 (45″)	3/5 (45″)	3/5 (45″)	3/5 (45″)	3/5 (45″)	3/5 (45″)	3/10 (45″)	3/10 (45″)	1/10	1/10	1/10	2/15	2/10	2/10
	semi-squat jumps in vertical movement (L/R)	2/5 (45″)	2/5 (45″)	2/5 (45″)	3/5 (45″)	2/5 (45″)	2/5 (45″)	2/5 (45″)	2/5 (45″)	2/5 (45″)	3/5 (45″)	2/5 (45″)	2/5 (45″)	2/10 (1″)	2/10 (1″)	2/10 (1″)	1/10	2/10	2/10
	semi-squat jumps in horizontal movement (L/R)	1/5	2/5 (45″)	2/5 (45″)	2/5 (45″)	3/5 (45″)	3/5 (45″)	2/5 (45″)	2/5 (45″)	2/5 (45″)	2/5 (45″)	3/5 (1″)	3/5 (1″)	2/5 (1″)	4/5 (1″)	4/5 (1″)	2/10 (1″)	2/10 (1″)	2/10 (1″)
	leg to leg jumps	2/5	2/5	2/5	2/10	3/10 (45″)	3/10 (45″)	1/10 (45″)	2/10 (45″)	2/10 (45″)	3/10 (45″)	2/10 (45″)	2/10 (45″)	2/10 (45″)	2/10 (1’)	2/10 (1’)	3/10 (1’)	3/10 (1’)	4/10 (1’)
**Contacts—Unilateral jumps**		60	70	80	100	110	110	80	90	90	140	150	150	100	120	120	130	150	160
**Week**		1			2			3			4			5			6		
**Training**		1	2	3	4	5	6	7	8	9	10	11	12	13	14	15	16	17	18
**Total number of surface contact**		**120**	**130**	**135**	**155**	**165**	**160**	**125**	**135**	**130**	**180**	**185**	**185**	**125**	**135**	**135**	**150**	**170**	**180**

**Table 2:** Repeated sprint ability training experimental group’s 6 week training program.

	1. Week	2. Week	3. Week	4. Week	5. Week	6. Week
**1. Training**	2 × 6 × 20 m 12 sprints	3 × 6 × 20 m 18 sprints	2 × 8 × 20 m 16 sprints	3 × 8 × 20 m 24 sprints	2 × 10 × 20 m 20 sprints	3 × 10 × 20 m 30 sprints
**2. Training**	2 × 6 × 20 m 12 sprints	3 × 6 × 20 m 18 sprints	2 × 8 × 20 m 16 sprints	3 × 8 × 20 m 24 sprints	2 × 10 × 20 m 20 sprints	3 × 10 × 20 m 30 sprints
**3. Training**	2 × 6 × 20 m 12 sprints	3 × 6 × 20 m 18 sprints	2 × 8 × 20 m 16 sprints	3 × 8 × 20 m 24 sprints	2 × 10 × 20 m 20 sprints	3 × 10 × 20 m 30 sprints
	36 sprints 240 m Total = 720 m	54 sprints 360 m Total = 1080 m	48 sprints 320 m Total = 960 m	72 sprints 480m Total = 1440 m	60 sprints 400 m Total = 1200 m	90 sprints 600 m Total = 1800 m
Total = 360 sprints, 7200 m						

**Table 3:** Results of univariate analysis in tests for estimation of motor and repeated sprint abilities of two experimental groups after repeated measurements.

	Plyometric		RSA		“Group”			“Time”			“Interaction T/G”		
Variables	Initial	Final	Initial	Final	F	P	Partial Eta-Squared	F	P	Partial Eta-Squared	F	P	Partial Eta-Squared
CMJ (cm)	39.94 ± 4.36	43.72 ± 4.02	39.96 ± 5.11	40.86 ± 5.20	0.72	0.40	0.02	24.116	0.00	0.47	9.11	0.00	0.25
RJ (cm)	33.92 ± 3.60	35.44 ± 3.55	34.87 ± 4.74	35.88 ± 4.15	0.22	0.63	0.00	3.935	0.05	0.12	0.15	0.69	0.00
SP5 m (s)	1.11 ± 0.07	1.11 ± 0.05	1.13 ± 0.06	1.12 ± 0.08	0.21	0.65	0.00	0.06	0.81	0.00	0.33	0.56	0.01
SP10 m (s)	1.84 ± 0.05	1.84 ± 0.06	1.88 ± 0.07	1.86 ± 0.09	1.27	0.26	0.04	0.19	0.66	0.00	0.32	0.57	0.01
SP25 m (s)	3.71 ± 0.06	3.7 ± 0.08	3.73 ± 0.11	3.68 ± 0.16	0	0.96	0.00	2.76	0.10	0.09	0.84	0.36	0.03
MAG20 y (s)	4.68 ± 0.13	4.87 ± 0.32	4.73 ± 0.17	4.75 ± 0.22	0.23	0.63	0.00	4.52	0.04	0.14	2.45	0.12	0.08
RSAb	3.78 ± 0.08	3.84 ± 0.16	3.74 ± 0.11	3.82 ± 0.13	0.55	0.46	0.02	8.37	0.00	0.23	0.15	0.69	0.00
RSAp	3.97 ± 0.10	3.99 ± 0.15	3.96 ± 0.14	3.99 ± 0.15	0.01	0.91	0.00	1.26	0.27	0.04	0.06	0.80	0.00
RSA%Sdec	4.98 ± 3.17	3.88 ± 1.48	5.8 ± 0.08	4.47 ± 2.23	1.01	0.32	0.03	5.57	0.02	0.17	0.04	0.83	0.00
RSA_La	13.07 ± 2.48	12.12 ± 2.06	14.77 ± 2.28	13.99 ± 3.07	4.42	0.04	0.14	3.127	0.08	0.10	0.01	0.89	0.00
RSA_RPE	7.33 ± 1.49	6.06 ± 1.48	8 ± 1.08	6.84 ± 1.28	2.92	0.09	0.10	16.94	0.00	0.39	0.03	0.84	0.00

**Legend**: CMJ—countermovement jump; RS—repeated jumps; SP5 m—sprint 5 m; SP10 m—sprint 10 m; SP25 m—sprint 25 m; MAG 20y—20 yards; RSAb—the best sprint in repeated straight-line sprint test; RSAp—average of sprints in repeated straight-line sprint test; RSA%Sdec—percentage of sprint decrease in repeated straight-line sprint test; RSA_La—concentration of lactates after repeated straight-line sprint test; RSA_RPE—subjective load estimation after repeated straight-line sprint test.

**Table 4:** Results of univariate analysis in tests for estimation of functional abilities of two experimental groups.

	Plyometric		RSA		″Group″			″Time″			″Interaction T/G″		
Variables	Initial	Final	Initial	Final	F	P	Partial Eta-Squared	F	P	Partial Eta-Squared	F	P	Partial Eta-Squared
VO_2_max (mL/kg/min)	55.16 ± 3.79	55.29 ± 4.72	55.2 ± 6.26	55.78 ± 5.28	0.02	0.88	0.00	0.30	0.58	0.01	0.11	0.73	0.00
Vmax (km/h)	16.6 ± 1.22	16.76 ± 1.01	16.61 ± 1.27	17.23 ± 1.08	0.32	0.57	0.01	11.1	0.00	0.30	3.67	0.06	0.12
vVO_2_max (km/h)	16.23 ± 0.90	16.33 ± 0.95	16.42 ± 1.13	16.61 ± 1.08	0.43	0.51	0.01	1.04	0.31	0.03	0.10	0.74	0.00
FSmax (bpm)	193.38 ± 8.48	192.53 ± 8.45	193.98 ± 7.96	190.07 ± 9.07	0.15	0.70	0.00	6.17	0.01	0.19	2.3	0.14	0.08
FSpeak_RSA (bpm)	188.07 ± 9.29	183.07 ± 10.25	189.39 ± 7.79	179.61 ± 8.39	0.11	0.73	0.00	23.8	0.00	0.49	2.48	0.12	0.09
FS1 min (bpm)	157.13 ± 13.37	152.73 ± 10.25	158.64 ± 14.11	148 ± 12.39	4.42	0.04	0.14	3.12	0.08	0.10	0.01	0.89	0.00

**Legend**: VO_2_max—maximum oxygen uptake; vmax—maximum speed of treadmill; vVO_2_max—maximum speed of treadmill at maximum oxygen uptake; FSmax—maximum heart frequency; FSpeak_RSA—peak heart frequency during repeated straight-line sprint test; FS1 min—heart rate frequency 1 min after repeated straight-line sprint test.
